# TNC upregulation promotes glioma tumourigenesis through TDG-mediated active DNA demethylation

**DOI:** 10.1038/s41420-024-02098-w

**Published:** 2024-08-01

**Authors:** Hongyu Xu, Shengrong Long, Chengshi Xu, Zhengwei Li, Jincao Chen, Bin Yang, Yongze He, Ziyue Xu, Zhiqiang Li, Wei Wei, Xiang Li

**Affiliations:** 1 Brain Research Center, Zhongnan Hospital of Wuhan University, Wuhan, China; 2 Department of Neurosurgery, Zhongnan Hospital of Wuhan University, Wuhan, China; 3 Frontier Science Center for Immunology and Metabolism, Wuhan University, Wuhan, China; 4 Medical Research Institute, Wuhan University, Wuhan, China; 5 Sino-Italian Ascula Brain Science Joint Laboratory, Zhongnan Hospital of Wuhan University, Wuhan, China

**Keywords:** Cancer genomics, CNS cancer, DNA methylation, DNA methylation

## Abstract

Gliomas represent the most predominant primary malignant tumor in central nervous system. Thymine DNA glycosylase (TDG) is a central component in active DNA demethylation. However, the specific mechanisms of TDG-mediated active DNA demethylation in gliomas remain unclear. This research indicates TDG expression is overexpressed in gliomas and correlated with poor prognosis. TDG knockdown suppressed the malignant phenotype of gliomas both in vitro and vivo. Notably, RNA-seq analysis revealed a strong association between TDG and tenascin-C (TNC). ChIP-qPCR and MeDIP-qPCR assays were undertaken to confirm that TDG participates in TNC active DNA demethylation process, revealing decreased DNA methylation levels and elevated TNC expression as a result. Silencing TNC expression also suppressed the tumor malignant phenotype in both in vitro and in vivo experiments. Additionally, simultaneous silencing of TNC reduced or even reversed the glioma promotion caused by TDG overexpression. Based on our findings, we conclude that TDG exerts an indispensable role in TNC active DNA demethylation in gliomas. The DNA demethylation process leads to alternations in TNC methylation levels and promotes its expression, thereby contributing to the development of gliomas. These results suggest a novel epigenetic therapeutic strategy targeting active DNA demethylation in gliomas.

## Introduction

Glioma, arising from glial cells, is the most prevalent intracranial malignancy [1]. Its annual incidence rate is 5–6/100,000, gliomas exhibit a significantly higher recurrence rate compared to other intracranial tumors, resulting in a generally poor prognosis, especially in glioblastoma multiforme (GBM) [2, 3]. Despite complete surgical resection and radio-chemotherapy, the mean survival time of GBM patients was only 14.6 months, with less than 5% of patients surviving beyond 3 years [4, 5]. Therefore, exploring the biological origins of gliomas and searching for potential diagnostic and therapeutic targets have always been the research focus in this field [6].

Specific genetic information and specific environmental factors are decisive in the occurrence of the vast majority in tumors [7, 8]. Epigenetics regulates the interaction between genes and the environment without changing the DNA sequence, achieving hereditary changes in gene expression, involving processes such as DNA methylation and histone modification [9, 10]. This regulatory network acts as a link between the environment and genetic information, primarily controlling gene expression through DNA methylation and histone modification, playing a crucial role in normal physiology and disease progression [11, 12]. Therefore, studying the regulation of DNA methylation is crucial for understanding the pathogenesis of tumors. DNA methylation is catalyzed by DNA methyltransferase (DNMT) family and uses S-adenosylmethionine as the methyl donor [13]. This process includes maintenance methylation and de novo methylation, mediated by the DNMT family (DNMT1 and DNMT3A, DNMT3B) [14]. Recent studies have found that DNA methylation in animal cells is reversible [15]. However, abnormalities in this mechanism might lead to disturbances in DNA methylation, thereby promoting the onset of malignant tumors and other diseases [16]. Understanding the enzymes related to DNA demethylation and their regulatory mechanisms in tumors is vital for clarifying the epigenetic regulatory mechanisms, the pathogenesis of tumors, and for finding potential therapeutic strategies. Although most research has focused on the ten eleven translocation (TET) family, studies on thymine DNA glycosylase (TDG) are relatively limited [17, 18]. TDG was originally discovered as a mismatch-specific DNA glycosidase, initially in HeLa cells [19]. TDG not only plays a key role in DNA repair but also participates in active DNA demethylation and gene transcription regulation, possibly serving as an instrumental contributor for cancer genesis. The upregulation of TDG expression can interact through transcription factors or transcriptional co-activators to moderate the expression of oncogenes, which may equally lead to genomic destablization and malignant tumourigenesis [20, 21]. Thus, TDG-mediated regulation of DNA demethylation modification holds a non-negligible relevance for cancer development.

Tenascin-C (TNC) is an integral part of the extracellular matrix exhibiting variable spatial and temporal distribution patterns during the entire life span [22, 23], During the development stage, TNC is expressed in neural and non-neural tissues at multiple sites involved in cell turnover, tissue remodeling and epithelial-mesenchymal interactions. TNC reaches its peak expression during embryonic and early postnatal development and then declines markedly with age [24, 25]. While its expression is typically low in most adult normal tissues, it elevates in certain contexts, such as stem cell niches, tendons, inflammation, and injury sites [26–28]. Importantly, abnormally high expression of TNC correlates with initiation and progression towards multitude of diseases, including atherosclerosis, thrombosis, heart failure, and cancer [29–31]. In recent years, numerous studies have started to focus on the relationship between TNC and malignant tumors, especially its impact on tumor progression, invasion, and metastasis, as well as its association with poor prognosis [32–35]. These studies offer crucial insights into the role of TNC in malignant tumors and its potential clinical implications. For glioma, a highly aggressive brain tumor, the current understanding of TNC’s role and associated mechanisms involved is still limited. Specifically, the expression patterns of TNC in gliomas, how it influences the development and invasive behavior of the tumor, and its relationship with molecular mechanisms such as DNA methylation, remain unanswered. Additional research is required to fully comprehend the contribution of TNC to glioma and its potential as a therapeutic target.

In this study, we first explored the distinct expression of TDG in glioma tissues as opposed to normal tissues and pro-oncogenic function. We delved further into understanding how TDG-mediated active DNA methylation influences TNC gene expression and explore TNC association with tumor tumourigenesis and immune microenvironment in glioma. Our aim was to refine a epigenetic regulatory mechanism in gliomas, particularly emphasizing the pivotal role of active DNA demethylation. This research highlights the involvement of active DNA methylation in glioma pathogenesis and points to novel therapeutic targets for DNA methylation regulation.

## Results

### Upregulation of TDG expression in glioma tissue associated with glioma grade and poor outcome

**Fig. 1:**
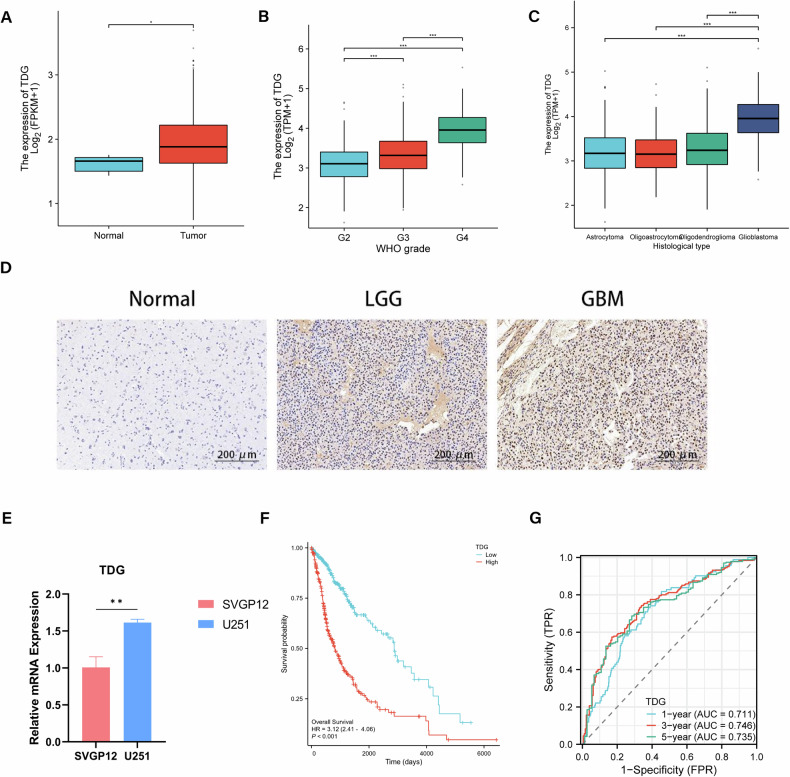
Upregulation of TDG expression in glioma tissue associated with glioma grade and poor outcome. **A**, **B** TDG mRNA expression in gliomas with normal brain tissues and different WHO grades from the TCGA database. **C** TDG mRNA expression levels of different histological subtypes were compared in gliomas from the TCGA database. **D** Representative IHC images of TDG in gliomas with different histological grades and NBT (200 µm). **E** The mRNA expression of TDG in SVGP12 and U251 cell lines were detected by RT-qPCR. **F** Kaplan-Meier survival curve of patients with glioma from the TCGA database stratified by TDG expression. **G** The receiver operator characteristic curve analysis of TDG utilizing the TCGA database.

RNA sequencing data from the TCGA database was used to study TDG expression levels in paraneoplastic tissues and glioma tissues of different grades, and patient characteristics are summarized in Table 1. TDG expression levels were elevated in glioma tissues versus normal brain tissues (Fig. 1A). Given the heterogeneity of different grades of gliomas, we compared the expression levels of TDG in different grades gliomas and observed higher TDG expression levels in high-grade gliomas, the expression level of TDG was upregulated and positively correlated with the tumor grade (Fig. 1B). It was also observed that TDG was most expressed in glioblastomas compared to other histological subtypes (Fig. 1C). Using a series of clinical glioma specimens, we investigated the TDG protein expression levels in 5 LGG, 5 GBM and 5 normal brain tissues by IHC, as well as mRNA expression levels of TDG in the SVGP12 and U251 cell lines by qPCR (Fig. 1D, E). We found that TDG has the same expression characteristics at the protein level in glioma tissues. With Kaplan-Meier survival curve analysis, we observed an overall survival that was considerably shorter in the TDG high-expression group (*n* = 350) than in the TDG low-expression group (*n* = 349), which portends a poor prognosis for glioma patients (Fig. 1F). We assessed the prognostic ability of TDG on glioma patients with ROC curves, which showed that the AUC of TDG for predicting one-year, three-year and five-year survival of patients was 0.711, 0.746, and 0.735, respectively (Fig. 1G). The results indicate that high levels of TDG expression in glioma patients is accompanied by a poor prognosis.

**Table 1:** Clinical information of glioma patients in TCGA database.

Characteristics	Low expression of TDG	High expression of TDG	*P* value
*n*	349	350	
WHO grade, *n* (%)			<0.001
G2	160 (25.1%)	64 (10%)	
G3	129 (20.3%)	116 (18.2%)	
G4	17 (2.7%)	151 (23.7%)	
IDH status, *n* (%)			<0.001
WT	62 (9%)	184 (26.7%)	
Mut	284 (41.2%)	159 (23.1%)	
1p/19q codeletion, *n* (%)			<0.001
Non-codel	238 (34.4%)	282 (40.8%)	
Codel	110 (15.9%)	62 (9%)	
Gender, *n* (%)			0.974
Female	149 (21.3%)	149 (21.3%)	
Male	200 (28.6%)	201 (28.8%)	
Age, *n* (%)			<0.001
≤60	305 (43.6%)	251 (35.9%)	
>60	44 (6.3%)	99 (14.2%)	
TNC, *n* (%)			<0.001
Low	235 (33.6%)	114 (16.3%)	
High	114 (16.3%)	236 (33.8%)	
OS event, *n* (%)			<0.001
Alive	268 (38.3%)	159 (22.7%)	
Dead	81 (11.6%)	191 (27.3%)	
Race, *n* (%)			0.335
Asian	4 (0.6%)	9 (1.3%)	
White	320 (46.6%)	320 (46.6%)	
Black or African American	18 (2.6%)	15 (2.2%)

### The suppression of TDG leads to inhibited proliferation, migration, invasion, and cell cycle progression

**Fig. 2:**
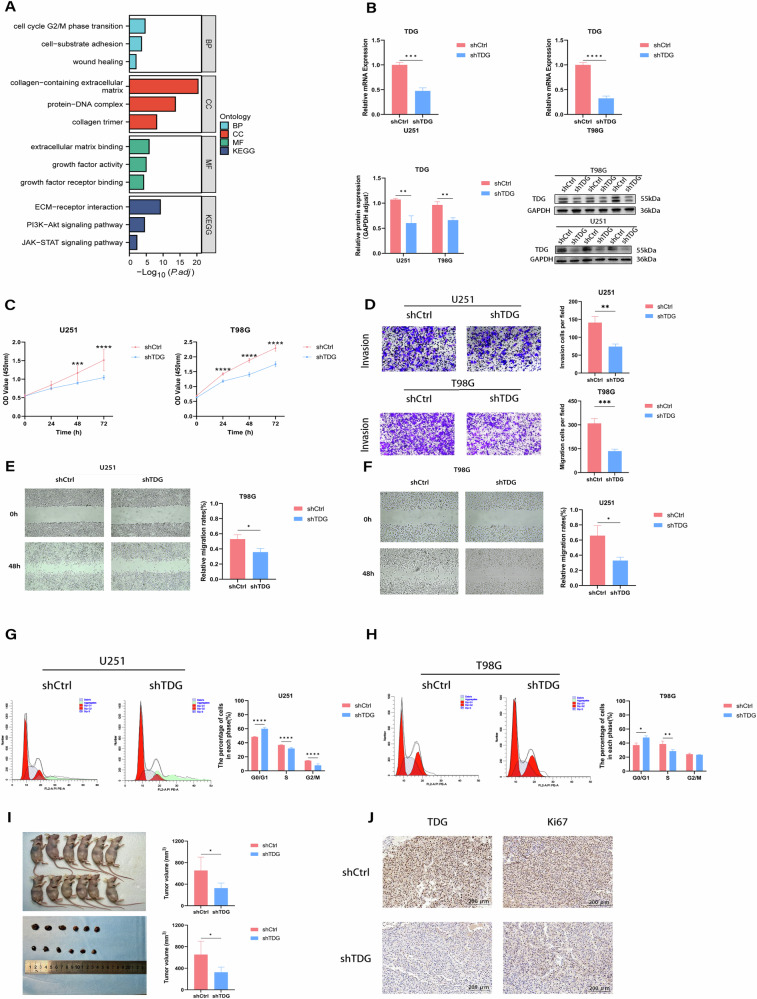
shTDG suppressed the cell proliferation, migration, invasion, and cell cycle progression of GBM cells in vivo and in vitro. **A** Differential genes GO and KEGG pathway analysis based on TCGA RNA-seq. **B** The mRNA and protein levels to detect cell transfection efficiency. **C** CCK8 assays show cell growth in U251 and T98G cell lines infected with shCtrl and shTDG. **D** Transwell assays were used to detect the invasive ability of U251 and T98G cell lines infected with shCtrl and shTDG. **E**, **F** Cell migration was measured by a wound healing assay. **G**, **H** Flow cytometry analysis for cell cycle distribution in U251 and T98G cell lines infected with shCtrl and shTDG. **I**, **J** shCtrl and shTDG infected U251 cells were injected into nude mice. The tumors weight and volume in mice are shown to have undergone alteration. The representative IHC images of xenograft tumors are shown.

To elaborate on the potential oncogenic function of TDG in gliomas, we screened high- and low-expressing glioma tissues for differentially expressed genes (DEGs) and enriched for GO and KEGG pathways using TCGA mRNA sequencing data. It was observed that the DEGs are primarily engaged in the cell cycle, wound healing, growth factor activity and PI3K-Akt signaling pathways (Fig. 2A). Notably, these pathways are closely implicated in the malignant phenotype of gliomas. Subsequently, the glioma cells transfected with TDG knockdown vector and control vector were used to validate the carcinogenic effects of TDG on gliomas in vivo and in vitro, respectively. RT-qPCR and protein blotting were used to check the alterations in TDG expression (Fig. 2B). The CKK8 assay revealed that silencing TDG severely inhibited the proliferation of glioma cells (Fig. 2C). Wound-healing and transwell assays verified that silencing TDG appeared to inhibit glioma cell migration and invasion (Fig. 2D, E, F). Knockdown of TDG-induced G1/G0 arrest in T98G and U251 cells, as revealed by flow cytometry (Fig. 2G, H). In summary, the present evidence reveals that TDG silencing inhibits glioma proliferation, migration, invasion and cell cycle progression in vitro.

### TDG suppressed glioma growth in vivo

In the following, to prove the impact of TDG on the regulation of glioma growth in vivo. Stable U251 cell lines, with or without TDG silencing, were subcutaneously injected into nude mice, resulting in the generation of tumors. After 16 days, TDG-silenced cells generated significantly slower tumor growth compared to control cells by the contrast in tumor volume and weight. IHC was conducted on paraffin-embedded specimens of xenografts to monitor changes in protein levels of Ki-67 and TDG between treatment groups, which were consistent with the in vitro results (Fig. 2I, J).

### TDG functions as a DNA demethylase by binding to TNC and facilitating TNC demethylation

**Fig. 3:**
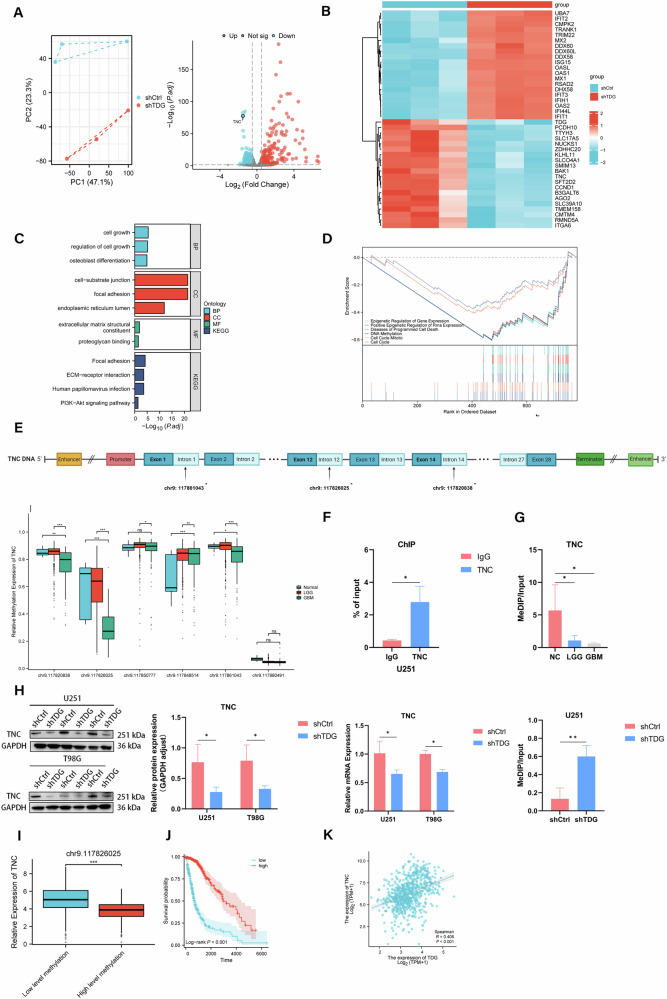
TDG collaborates with TNC to facilitate active DNA demethylation. **A** DEGs were identified in TDG knockdown or negative control stably transfected U251 cells and PCA. **B** DEGs heatmap. **C**, **D** GO and KEGG pathway analysis and GSEA based on DEGs from RNA-seq. **E** TNC DNA methylation changes and sites in the TCGA and GEO databases. **F** ChIP assays were performed on the TDG and TNC binding regions. **G** TNC 5mC changes in paraneoplastic and glioma tissues measured by MeDIP-qPCR. **H** The inhibition efficiency of TNC expression alteration was detected by RT-qPCR and western blotting. TNC 5mC changes in TDG knockdown and control U251 cells measured by MeDIP-qPCR. **I** TNC methylation levels in relation to mRNA expression levels from TCGA database. **J** Relationship between methylation level of TNC at chr9:117826025 and survival probability by Kaplan-Meier curve. **K** The correlation between TDG and TNC from TCGA database.

To investigate the crucial role played by TDG-mediated active DNA demethylation modification in gliomas and the underlying mechanisms of action. We performed RNA sequencing using TDG knockdown or negative control stably transfected U251 cells, to explore the downstream target genes of active DNA demethylation involved in TDG. A total of 783 differential genes were detected following the knockdown of TDG in glioma cells, and principal components analysis was conducted (Fig. 3A). The numerical heatmap below illustrates the altered gene expression upon TDG knockdown (Fig. 3B). Subsequently, we observed that DEGs are mainly involved in processes such as cell growth, cell-matrix junctions, structural components of the extracellular matrix and ECM-receptor interactions by GO and KEGG analyses (Fig. 3C). Similarly, the gene set enrichment analysis (GSEA) demonstrated that TDG silencing significantly affects the cell cycle and programmed cell death, as well as the epigenetic regulation of genes, particularly related to DNA methylation levels (Fig. 3D). Excitingly, tenascin-C (TNC), a crucial ECM glycoprotein involved in the process of cell growth, cell-substrate junction and ECM-receptor interaction, was markedly downregulated in TDG knockdown glioma cells. Therefore, we centered our research on the interaction between TDG and TNC. Initial analysis of 589 glioma samples from the TCGA and GEO databases revealed decreased DNA methylation levels of TNC at three sites: chr9: 117820838, chr9: 117826025, and chr9: 117861043. These sites are located within the gene’s intronic region, with the most notable decrease observed at chr9:117826025 (Fig. 3E). To verify that the downregulation of methylation levels of TNC in gliomas is mediated by active DNA demethylation of TDG, we confirmed by ChIP- qPCR assay that TDG was able to combine with the chr9: 117826025 position of TNC, participate in the DNA demethylation process of TNC (Fig. 3F). Subsequently, we performed MeDIP analysis with TDG knockdown and control U251 cells, as well as checked the alterations in TNC expression using RT-qPCR and protein blotting, indicating that TDG knockdown greatly enhanced the DNA methylation level of TNC, suppressing TNC expression in glioma cells (Fig. 3H). In addition, we carried out MeDIP analysis employing glioma and paraneoplastic tissues from 4 GBM and 4 LGG patients, the equivalent downturn in TNC methylation levels was observed in GBM (Fig. 3G). We meanwhile detected a remarkable negative correspondence between TNC hypomethylation level and TNC expression in glioma patients (Fig. 3I). Kaplan-Meier survival curve analysis revealed that patients with low TNC DNA methylation levels had significantly shorter survival than patients with high levels of DNA methylation expression (Fig. 3J). As the main participant of DNA active demethylation, the expression of TDG in glioma is positively correlated with TNC (Fig. 3K).

### TNC upregulation is linked to the tumor immune microenvironment and poor outcomes in glioma

**Fig. 4:**
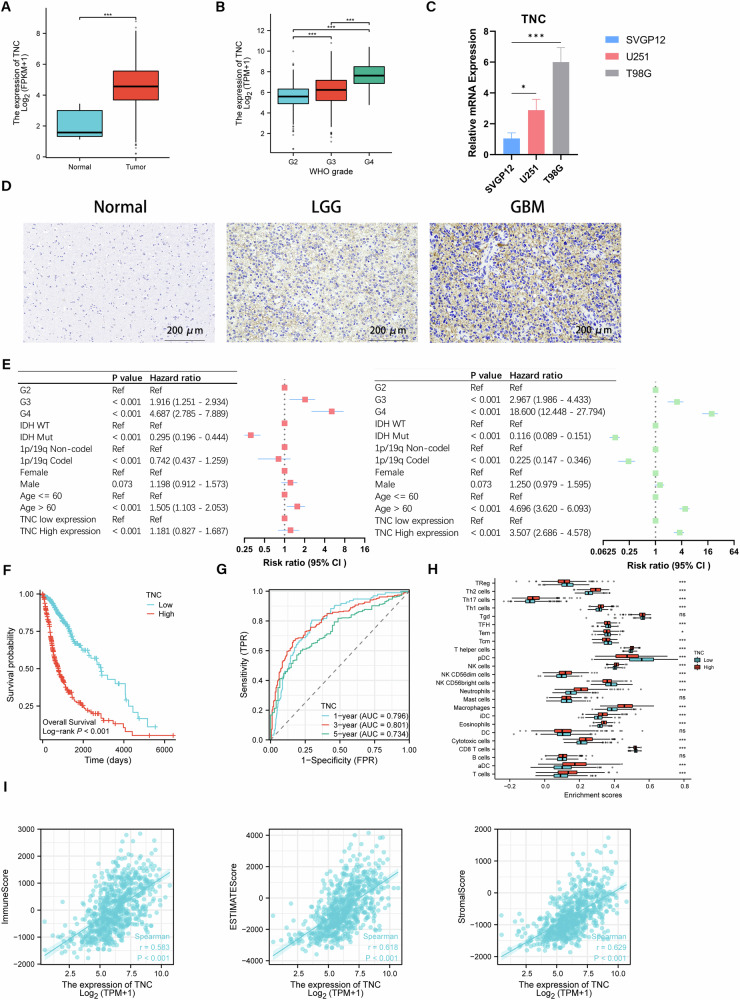
TNC upregulation is linked to the tumor immune microenvironment and poor outcomes in glioma. **A**, **B** TNC mRNA expression in gliomas with normal brain tissues and different WHO grades from the TCGA database. **C** The mRNA expression of TNC in SVGP12, U251 and T98G cell lines were detected by RT-qPCR. **D** Representative IHC images of TNC in gliomas with different histological grades and NBT (200 µm). **E** Univariate and multivariate Cox proportional hazards model using TCGA database. **F** Kaplan-Meier survival curve of patients with glioma from the TCGA database stratified by TNC expression. **G** Prognostic nomogram to predict 1-, 3-, and 5-year survival probability for patients with gliomas. **H, I** Correlation between TNC expression and 24 kinds of immune infiltration cells in TCGA database. The risk score was significantly correlated with the immune scores, stromal scores and ESTIMATE scores, respectively.

The expression level of TNC was particularly elevated in glioma tissue compared to normal brain tissue, and also had a positive correlation with tumor grade in the TCGA database (Fig. 4A, B). We also investigated the TNC protein expression levels in five LGG, five GBM and five normal brain tissues by IHC, as well as mRNA expression levels of TNC in the SVGP12, U251, and T98G cell lines by RT-qPCR. We found the same expression characteristics in glioma tissues (Fig. 4C, D). To further explore the clinical relevance of TNC and forecast its prognostic value in glioma patients, we categorized patients with glioma from TCGA data. We observed that the overall survival of the high TNC expression group was considerably shorter than that of the low TNC expression group. (Fig. 4F). The ROC results showed that the AUC of TNC for predicting 1-, 3- and 5-year patient survival was 0.796, 0.801, and 0.734, respectively (Fig. 4G). These results suggest that abnormally high levels of TNC expression have a credible predictive value for poor prognosis in glioma patients. Afterwards, we constructed a univariate and multivariate Cox proportional hazards model using six prognostic factors to explore the relationship between TNC, clinical characteristics and overall survival (Fig. 4E). To further characterize the effect of TNC on the tumor microenvironment. The relationship between TNC and 24 tumor-infiltrating immune cells was analyzed by applying the ssGSEA method with two groups of high and low TNC expression. The findings revealed that TNC expression was significantly positively correlated with macrophages, eosinophils, and neutrophils, etc., and closely negatively correlated with plasmacytoid dendritic cells and NK cells, etc. (Fig. 4H). Using the ESTIMATE method, we determined the relevance of TNC in glioma microenvironment. Pearson correlation analysis identified a direct association among TNC expression, immune score, matrix score and estimate score (Fig. 4I).

### TNC knockdown inhibits glioma cell proliferation, migration, invasion, and cell cycle in vitro and vivo

**Fig. 5:**
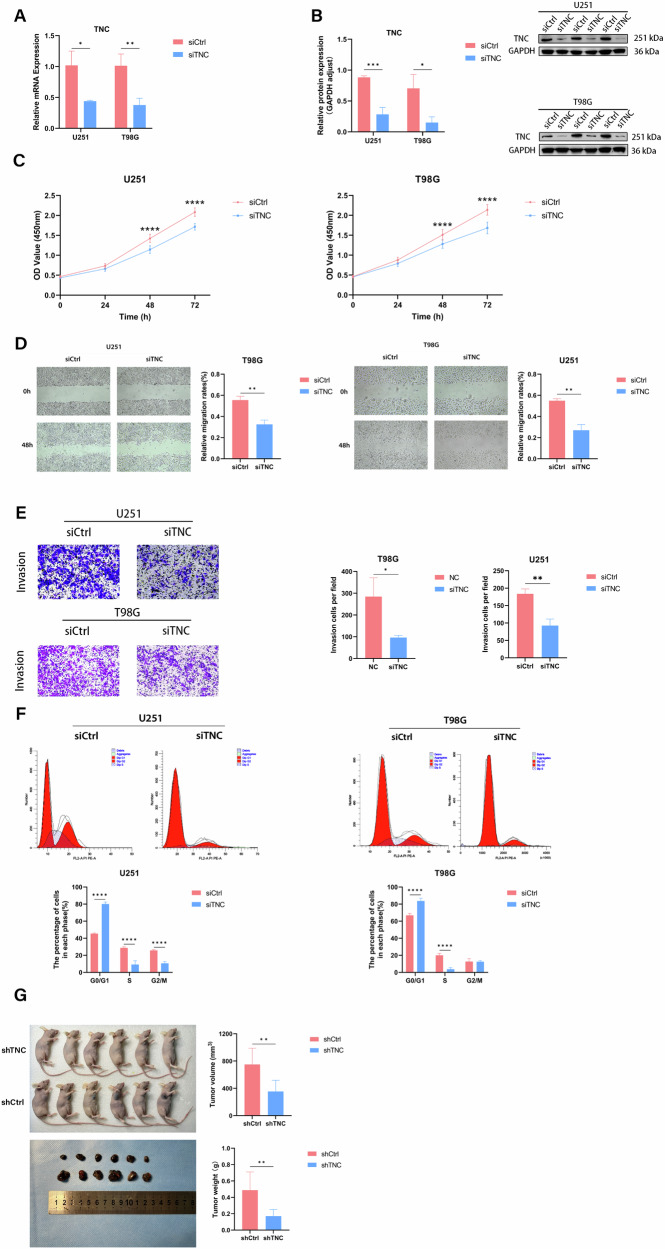
TNC promotes GBM cell proliferation, migration, invasion, and cycle progression in vivo and in vitro. **A**, **B** TNC-siRNA effectively inhibits the mRNA and protein expression of TNC in U251 and T98G cells. **C** CCK8 assays show cell growth in U251 and T98G cell lines infected with siCtrl and siTNC. **D** Cell migration was measured by a wound healing assay. **E** Transwell assays were used to detect the invasive ability of U251 and T98G cell lines infected with siCtrl and siTNC. **F** Flow cytometry analysis for cell cycle distribution in U251 and T98G cell lines infected with siCtrl and siTNC. **G** Tumor weight and volume alteration in mice. The representative IHC images of xenograft tumors are shown.

To investigate further the oncogenic role of TNC in gliomas, we designed siRNA sequences against TNC and estimated its knockdown efficiency in glioma cells for subsequent experiments (Fig. 5A, B). Then, CCK-8, wound healing, invasion assays, and flow cytometry were conducted to investigate whether downregulation of TNC could alter glioma cell proliferation, migration, invasion ability, and inhibit the cell cycle. Knocking down TNC led to a dramatic inhibition of the proliferation, migration and invasion ability of U251 and T98G cells (Fig. 5C, D, E). Flow cytometry results displayed that knockdown of TNC in T98G and U251 cells induced G1/G0 arrest (Fig. 5F). These observations imply that TNC is involved in the regulation of cell proliferation, migration, invasion and cell cycle progression. Further, we investigated the effects of TNC in vivo. The findings from the tumor xenograft trials proved that shTNC significantly suppressed the size of the transplanted tumors by comparing tumor volume and weight. These data are consistent with the results of in vitro (Fig. 5G).

### TDG promotes glioma progression by facilitating TNC demethylation

**Fig. 6:**
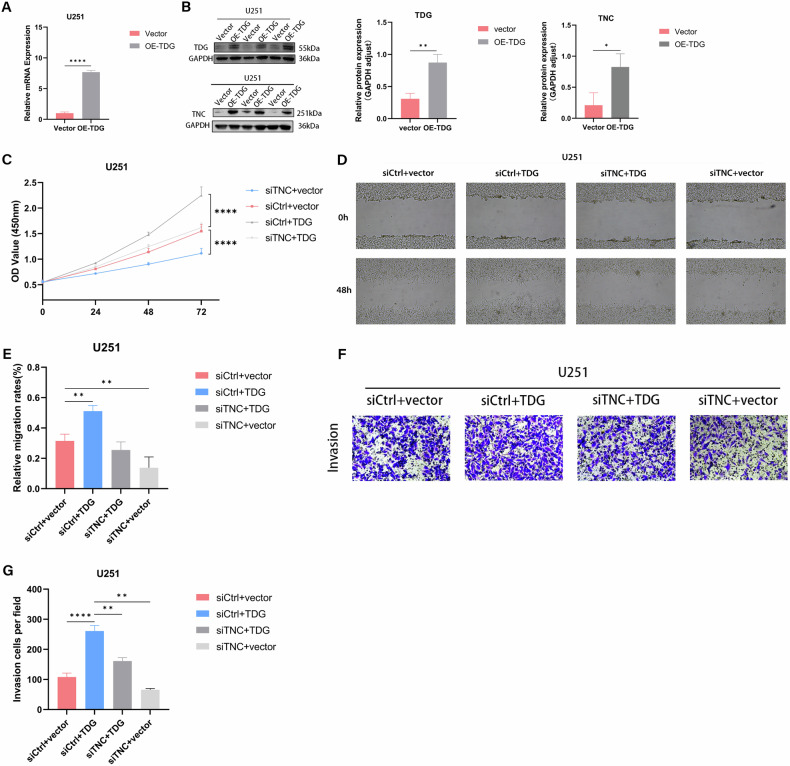
TNC partially reversed the effect of TDG in GBM cells. **A**, **B** The overexpression efficiency of TDG and TNC expression alteration was detected by RT-qPCR and western blotting. **C** CCK8 assay showing cell growth in U251 cells infected with TDG overexpression or the control (vector), and infected with siCtrl or siTNC. **D**, **E** Cell migration was measured by a wound healing assay. **F**, **G** Transwell assays were used to detect invasive ability in U251 cell lines.

To validate the involvement of TNC in the malignant phenotype of TDG-induced gliomas, we established stable cell lines by transfecting siTNC into TDG-overexpressing cells for the rescue assay. The findings from qPCR and Western blotting show an increase in both TDG mRNA and protein expression levels in TDG overexpressing cells. Furthermore, a noteworthy observation is the substantial upregulation of TNC protein expression levels in the U251 cell line with TDG overexpression (Fig. 6A, B). CCK-8 assay revealed that inhibition of TNC remarkably decreased the proliferation of glioma cells when TDG was overexpression (Fig. 6C). Transwell and wound healing assays demonstrate that the reduction of TNC amplifies invasion and migration when TDG is upregulated (Fig. 6D–G). These studies add to the evidence that TDG promotes the malignant progression of glioma by inducing the expression of TNC.

## Discussion

TDG removes new cytosine derivatives produced by TET enzymes during active DNA demethylation associated with genomic demethylation [36, 37]. This is crucial for active DNA demethylation, as highlighted by previous research [38, 39]. Alternatively, TDG can act on G/T or G/U mismatches, repairing bases that do not match G and initiating the base excision repair (BER) pathway. During active DNA demethylation, 5-fluorocytosine and 5-carboxylcytosine are removed through TDG-mediated excision, leading to the formation of base-free sites and consecutive DNA single-strand damage. These sites can then be changed into C bases through final repair via the BER pathway [40]. In most types of cancer, DNA methylation patterns are quite altered [41]. Recent evidence has highlighted that alterations in DNA methylation are involved in glioma ontogeny and can determine patient survival as a common biological event [42]. Dysregulated expression of TDG strongly influences the balance between enzymes in the DNA demethylation pathway and negatively affects genome stability and regulation of gene expression. Studies have demonstrated that TDG can act on DNMT3A to promote its ubiquitination degradation, triggering the imbalance between methylase and demethylase and leading to tumourigenesis [43, 44]. In contrast to previous reports, since decreased TDG expression induces the accumulation of 5-hydroxymethylcytosine, 5-cac and 5-fc, TDG has been suggested to serve as a protective component in malignant tumors [45, 46]. The TET and DNMT families have now been proven to modulate the transcriptional regulation of diverse target genes engaged in glioma pathogenesis [47]. For instance, the reduction of TET2 enzyme levels by promoter methylation in absence of TET2 mutation was reported recently in low-grade diffuse astrocytomas, suggesting that this represents an additional pathomechanism in IDH1- and IDH2-mutated low-grade gliomas [48]. Our research concentrates on the demethylation of TDG in gliomas, demonstrating that upregulation of TDG expression in glioma cells leads to a significant downregulation of TNC methylation levels, thereby promoting TNC expression. Ultimately, we elucidated the relationship between active demethylation and the malignant behavior of gliomas, providing ideas for the mechanistic investigation of gliomagenesis.

TNC is a member of the pathogenesis of glioma, interacting with several factors, most of which cause tumor progression. One example is an in vitro experiment demonstrating that the proliferation of brain tumor-initiating cells can be reduced by activated T cells, although this effect is attenuated by BTIC secretion of TNCs via exosomes [49]. The clinical significance of TNC for gliomas is widely recognized. A variety of monoclonal antibodies against TNC are now available and can also be combined with temozolomide, which can be targeted to fight gliomas [50, 51]. Distinct from numerous oncogenes, TNC is simultaneously involved in tumor angiogenesis, immunomodulation and EMT, which is the reason why TNC has attracted considerable attention in recent years. TNC is involved in and regulates glioma angiogenesis in two ways: on the one hand, TNC inhibits angiogenesis by blocking YAP signaling and endothelial cell behaviors through direct contact. On the other hand, TNC generates ephrin-B2 and a pro-angiogenic secretome in glioblastoma cells [51]. Besides angiogenesis, TNC also participates in vascular mimicry and transdifferentiation of glioma stem cells [52, 53].

**Fig. 7:**
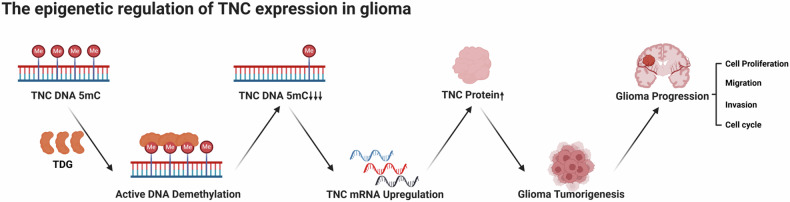
The epigenetic regulation of TNC expression in glioma. The expression of TNC is increased by TDG-mediated active DNA demethylation, which is a crucial process in the development of the malignant phenotype of glioma.

In our study, we initially revealed that TDG expression was significantly associated with the prognosis of glioma patients, which was also validated in clinical samples and human glioma cell lines. Meanwhile, we further delved into the specific mechanisms of TDG-mediated active DNA demethylation leading to the overexpression of TNC and promoting glioma tumourigenesis by RNA-seq, ChIP-qPCR, and MeDIP-qPCR. Our findings strongly implicate that TDG exerts a function similar to tumor-promoting factors, mediating the up-regulation of TNC expression, and is involved in glioma progression. Gliomas are characterized by a high degree of vascularization [51], TDG regulates TNC expression through active demethylation and we have identified the demethylation sites to fully elucidate this process (Fig. 7). Therefore, the focus of further research will be to determine whether TDG can directly or indirectly mediate the formation of glioma neovascularisation and whether it can also mediate the expression of TNC. Simultaneously, whether the active DNA demethylation mediated by TDG can interact with DNA methylase may collectively influence the occurrence of glioma.

Collectively, TDG is involved in the induction of TNC hypomethylation in GBM, which elevates TNC expression and significantly promotes the malignant phenotype of human glioma cells. In addition, our study also identified a tight connection between the upregulation of TNC and immune infiltration, which predicts that immune-targeted therapy against the tumor microenvironment in combination with TNC will become possible. Meanwhile, due to the high affinity in TNC and glioma neovascularization, whether active demethylation of TDG can be directly engaged in the formation of tumor neovascularization will be the emphasis in future research. Targeted action to suppress glioma neoangiogenesis may also serve as a potential diagnostic paradigm for glioma treatment in the foreseeable future. As a result, the role of TDG in epigenetic regulation to facilitate glioma development extends knowledge into the mechanisms of TDG-mediated demethylation regulation and translates into therapeutic strategies for GBM.

## Materials and methods

### Data download

RNA-seq and clinical data from glioma patients, including low-grade gliomas (LGG) and glioblastomas (GBM), were downloaded from the TCGA database (https://www.cancer.gov/tcga). A total of five glioma paraneoplastic tissues and 699 glioma tissues (excluding two patients with incomplete clinical information) were obtained for the purpose of conducting combined gene expression and clinical information analyses. A total of five glioma paraneoplastic tissues and 699 glioma tissues were used for the combined gene expression and clinical information analyses, and DNA methylation data from 582 of these glioma patients and seven paraneoplastic tissues (two cases from TCGA database, five cases from GEO database) were downloaded for the following analyses [54].

### RNA-sequencing analysis

Total RNAs were prepared from TDG-silenced U251 cells and appropriate negative control cells for quality control. The Agilent RNA 6000 Nano Kit and the Agilent Bioanalyzer 2100 (Agilent Technologies, USA) were employed in order to assess the integrity of the total RNA. 1ug of RNA was subsequently processed using the VAHTS Universal V8 RNA-Seq Library Prep Kit for Illumina (Vazyme NR605, China) for library preparation and Agilent Bioanalyzer 2100 for quality control, followed by Illumina sequencing. Mapping raw reads to the Homo sapiens reference genome using HISAT2 software [55]. Converting mapping reads to genomic features using FeactureCounts [56]. Differential expression analysis with the limma package [57] the genes with |logFC | > 0.5, adjusted *P* value < 0.05, were determined as DEGs. To assess the functional characteristics of the DEGs, KEGG and GO were used to perform enrichment analysis [58]. Functional items with an adjusted *P* value of less than 0.05 were identified as relevant.

### Tumor sample collection

Glioma and normal brain tissues were obtained from patients who underwent surgical resection at Zhongnan Hospital of Wuhan University with informed consent. Pathological grading of gliomas is diagnosed based on the 2021 WHO Classification of Tumors of the Central Nervous System [59]. None of the patients had received radiotherapy prior to surgery. Pathological analysis of the collected gliomas and paracancerous tissues was performed independently by two pathologists. A portion of the tissue was excised and fixed in paraformaldehyde for subsequent immunohistochemical analysis. The remaining tissue was stored in an ultra-low temperature refrigerator at −80°C.

### Real-time quantitative PCR (RT-qPCR)

Total RNA was obtained from the U251 and T98G cell lines and brain tissue samples using Takara RNAiso Plus (Takara Bio. Inc., Otsu, Shiga, Japan) following the manufacturer’s instructions. Reverse transcription of cDNA from 1 μg RNA was carried out by qPCR with HiScriptIIQ RT SuperMix kit (Vazyme Biotech). The 2-ΔΔCT method was used to process qPCR data [59]. Supplementary Table 1 lists the primer sequences used in this study.

### Cell culture

The human glioblastoma multiforme cell lines U251 and T98G, and the human astrocyte lines SVGP12 were purchased from ATCC and have undergone multiple Short Tandem Repeat (STR) analyses within the last 3 years to confirm identity. The U251, T98G and SVGP12 were cultivated in Dulbecco’s modified Eagle medium (DMEM, Servicebio) containing 10% fetal bovine serum (FBS, LONSERA) and 10 μl/ml penicillin–streptomycin (Biosharp) at 37 °C in a humidified chamber with 5% CO_2_.

### Cell transfection

TNC’s small interfering (si)RNA and RNA TransMate were obtained from RiboBio (Guangzhou, China). All plasmids mentioned in this paper were synthesized by Sangon Biotech (Shanghai, China). Sequence information is provided in Supplementary Table 1. Log-phase U251 and T98G cell lines were infected with lentiviral vectors encoding polyacrylamide for 48 h. The lentiviral infection efficiency on the cells was observed to be more than 85% by fluorescence microscopy before the cells were picked for further experiments.

### Immunohistochemistry (IHC)

All tissues were fixed, embedded and sectioned. Incubation with specific TDG (13370-1-AP, Proteintech Group) and TNC (ET1608-50, HUABIO) primary antibodies and poly-HRP-conjugated goat-anti-rabbit IgG, respectively. Sections were stained at room temperature and observed by light microscopy.

### Western blot

After lysing the cells with an iced IP lysate, protein concentrations were accurately measured using the BCA Protein Assay Kit (Beyotime Biotech). An equivalent protein from each sample underwent separation on a 4–20% ExpressPlus™ PAGE Gel and was transferred to a PVDF membrane (Bio-Rad Laboratory). The membranes were then blocked with western sealing solution for 1 h. After washing with TBST, the membranes were subsequently incubated with primary antibodies (1:1000) overnight at a temperature of 4 °C. The next day, membranes were incubated with HRP-labeled secondary antibody (Proteintech, Wuhan, China) for 1 h at RT. The signals were visualized using the enhanced chemiluminescence solution (ECL, Servicebio), with band intensities quantified via Image J software. Full and uncropped western blots were presented in Supplemental Material 1.

### CCK8 assay

The manufacturer’s protocol (A311-01, Vazyme Biotech, Nanjing, China) was followed to perform the CCK8 assay for evaluating cell proliferation. We spread 5000 cells per well evenly in a 96-well plate. After 6, 24, 48, and 72 h of incubation, the medium of each well was aspirated and 10% CCK8 solution was added 100 μl per well and cultured for 1 h at 37 °C protected from light. Absorbance was then read at 450 nm using a BioTek Synergy HT microplate reader.

### Wound healing assay

We cultured one million cells in six-well plates in order to achieve 90–100% fusion the next day. Scratches were created on the cell surface using a 10 µl disposable pipette tip. The cells were subsequently washed and cultivated with 2.5% FBS concentration medium. The area of the wound gap was calculated by ImageJ software at 0 and 48 h.

### Transwell invasion assay

The Corning Matrigel Invasion Chamber was employed to conduct the invasion assay. Transwells in 24-well plates were seeded with 40,000 cells per well. Add 100 µL serum-free medium to the upper chamber. 500 µL of complete medium supplemented with 10% serum was added to the lower chamber and incubated for 48 hours. At the end of the culture, the cells were fixed. Floating cells were gently wiped off with a cotton swab and stained with 0.5% crystal violet. Cell images were captured and processed with the ImageJ software.

### Cell cycle analysis

For cell cycle analysis, trypsin-digested cells were resuspended in PBS and then fixed overnight by adding 1 mL of ice-cold 75% ethanol. Subsequently exposed to freshly prepared PI staining solution at 25 °C for 0.5 h in the dark. The cell cycle populations were assessed by ModFit software.

### Chromatin immunoprecipitation (ChIP) assay

In summary, following a 6-min cross-linking with formaldehyde, glioma cells were subsequently quenched with 125 mM glycine for a further 6 min. DNA strands were fragmented into pieces ranging from 200 to 500 bp through sonication. The cross-linked protein-DNA complexes were immuno-precipitated using a TDG antibody (Proteintech Group, 13370-1-AP) and a control IgG antibody (ABclonal, AC011). The immunoprecipitated DNA was analyzed through qPCR. The primer sequences are presented in the Supplementary Table 1.

### Methylated DNA immunoprecipitation (MeDIP)

The entire genomic DNA was sonicated to DNA fragments between 200 bp and 1000 bp in length. Single-stranded DNA was generated by heat denaturation of one microgram of fragmented DNA. Immunoprecipitation was performed at 4 °C overnight using 1 μg anti-5mC antibody. Finally, DNA fragments bound to specific proteins were captured, eluted, and used for real-time PCR detection.

### Tumor xenograft experiments

The mice were randomly assigned to various groups and subjected to subcutaneous transplantation (*n* = 6). 2 million cells were injected subcutaneously into the left flank of each 4-week-old female BALB/c nude mice. The mice were euthanised 16 days after implantation, followed by volume measurement and weighing post tumor excision. Tumors were finally fixed in 4% paraformaldehyde. The tumor volume was calculated: volume (mm^3^) = (length × width^2^)/2.

### Bioinformatic analysis

The Kaplan-Meier curve was drawn with the survival and survminer packages in R software. The survival receiver operating characteristic (ROC) package was utilized to generate an ROC survival curve for patients with glioma obtained from TCGA databases. KEGG and GO enrichment analysis was carried out by employing the clusterProfiler package [60]. DEGs were analyzed using GESA 4.1.0 [58]. The pathways to be analyzed were obtained from the c2.cp.kegg.v7.0.symbols.gmt dataset in the Molecular Signature Database (MsigDB) and Reactome Knowledgebase (https://reactome.org) [61]. The results of the enrichment analysis were visualized using the ggplot2 package [62]. Heatmap visualization with the ComplexHeatmap package. CIBERSORT [63] and ESTIMATE [64] packages were employed to compute the ratio of various immune-infiltrating cells, immune scores, and stromal scores in patients diagnosed with glioma.

### Statistical analyses

Statistical analysis was conducted using R v.4.1.0 and GraphPad Prism v.9.0. To compare different indicators in the public data, the Wilcoxon rank-sum test and the Student’s t-test were used. To analyze the overall survival, we employed the Kaplan-Meier method. In addition, the R package “pROC” [65] was used for ROC curve analysis to predict overall survival. Univariate and multivariate Cox regression analyses were employed to identify autonomous prognostic factors. *P < 0.05 was considered statistically significant.

### Supplementary information

Original western blots

Supplementary Table 1

Cell line STR

## Data Availability

The data that support the findings of this study are available from the corresponding author upon reasonable request.
