# Supplementary figures and images for: TNC upregulation promotes glioma tumourigenesis through TDG-mediated active DNA demethylation
Source: Cell Death Discov. 2024 Aug 1;10:347. doi: 10.1038/s41420-024-02098-w (PMC11294444; doi:10.1038/s41420-024-02098-w)

**Supplemental Material** 1 Original western blots in this research.

Figure 2B


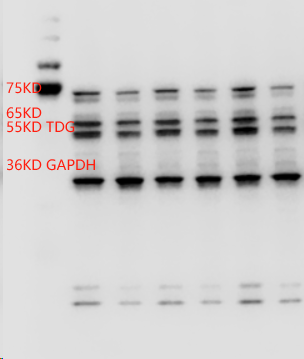

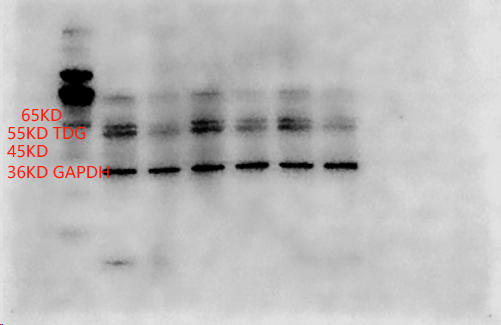


Figure 3H


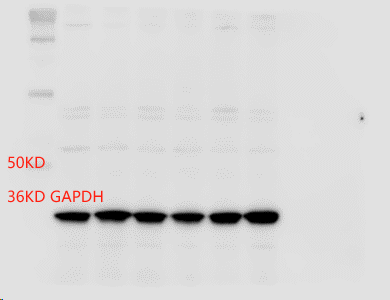

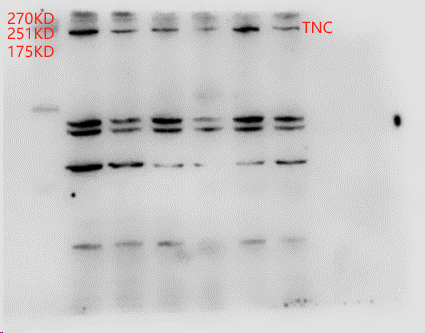


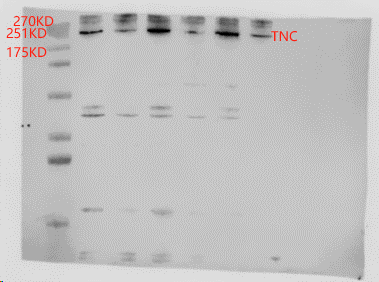

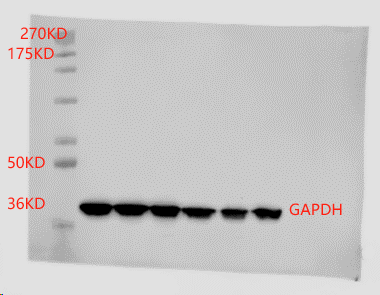


Figure 5B


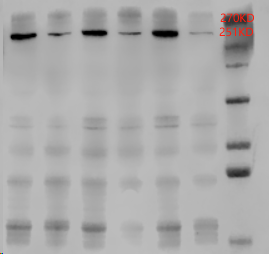

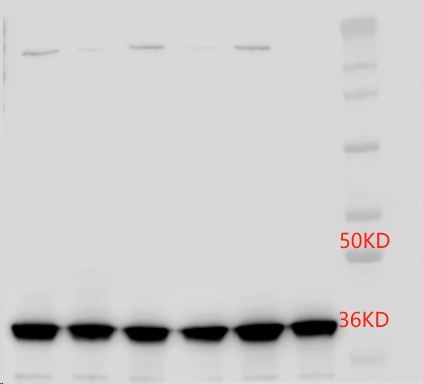


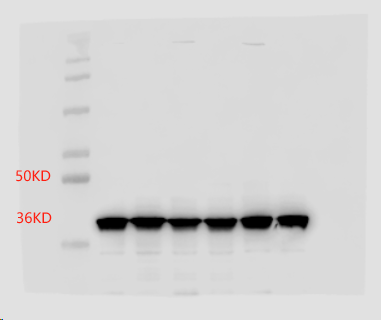

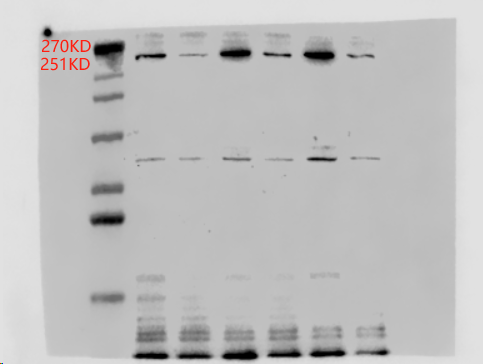

Supplement: Supplementary file 1 — Original western blots [file 41420_2024_2098_MOESM1_ESM.doc]
