# Supplementary material for: TNC upregulation promotes glioma tumourigenesis through TDG-mediated active DNA demethylation
Source: Cell Death Discov. 2024 Aug 1;10:347. doi: 10.1038/s41420-024-02098-w (PMC11294444; doi:10.1038/s41420-024-02098-w)
Supplement: Supplementary file 2 — Supplementary Table 1 [file 41420_2024_2098_MOESM2_ESM.docx]

**Supplementary Table 1** Primers, shRNAs and siRNAs sequences used in this research.

Primers used for RT-qPCR

| Gene symbol | Forward primer sequence (5′→3′) | Reverse primer sequence (5′→3′) |
| --- | --- | --- |
| TDG | ACGAGGGTGGTCTGGAAATG | GGATGGCAAATACACGGATAAAG |
| TNC | GTCTCTGGGGTTGTCTTACCG | CTTGCTGAAGGGAATAGCTGC |
| GAPDH | GGAGCGAGATCCCTCCAAAAT | GGCTGTTGTCATACTTCTCATGG |

Primers used for ChIP-qPCR and MeDIP-qPCR

| Gene symbol | Forward primer sequence (5′→3′) | Reverse primer sequence (5′→3′) |
| --- | --- | --- |
| TNC | ACGAATTCACTAATTCACTTCCTCC | TGGTAGGAGCTGATCCCAGT |

Sequences of siRNAs and shRNAs

| Gene symbol | Sequence (5'->3') |
| --- | --- |
| siTNC-1 | GGAGTACTTTATCCGTGTA |
| siTNC-2 | GTTACCTGCTGGTCTATGA |
| siTNC-3 | GCCACTCATTATACCATCA |
| shTDG | GAACGAAATATGGACGTTCAA |
| shNC | GAGCAGAAGCAGGTGCAGA |
| shTNC | GGAGTACTTTATCCGTGTATT |
