# Supplementary material for: TNC upregulation promotes glioma tumourigenesis through TDG-mediated active DNA demethylation
Source: Cell Death Discov. 2024 Aug 1;10:347. doi: 10.1038/s41420-024-02098-w (PMC11294444; doi:10.1038/s41420-024-02098-w)

Supplemental Material 2 Cell line STR in this research.

SVGP12 (Procell Life, CL-0233, RRID: CVCL_3797)


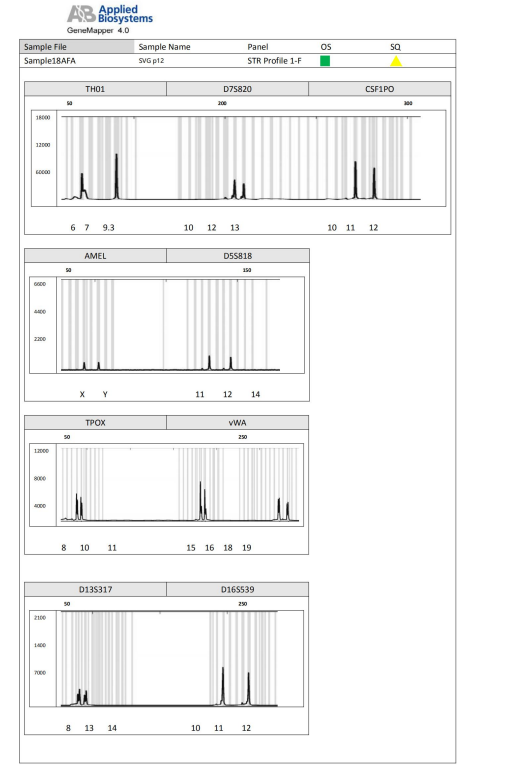


U251 (Procell Life, CL-0237, RRID: CVCL_0021)


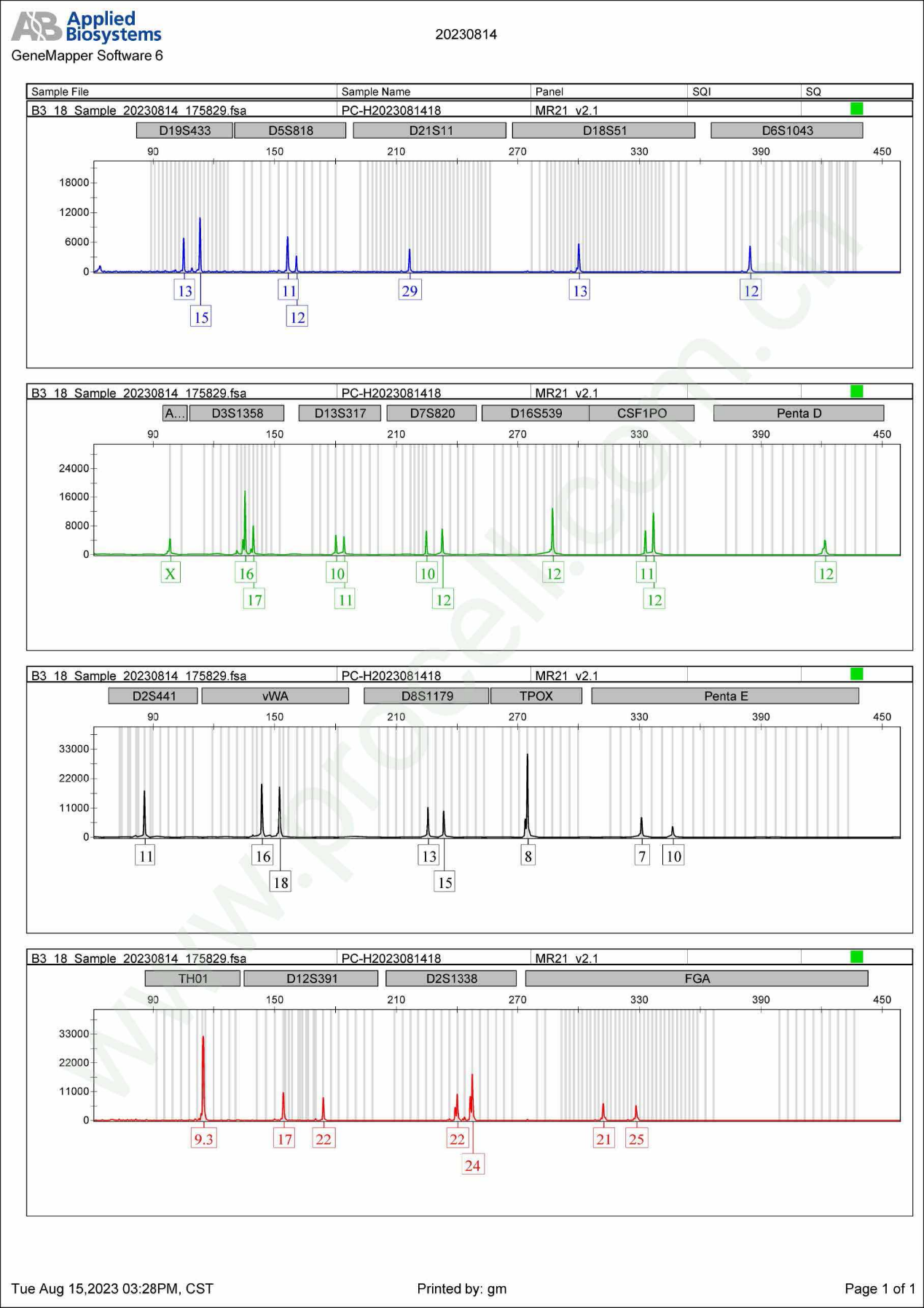


T98G (Procell Life, CL-0583, RRID: CVCL_0556)


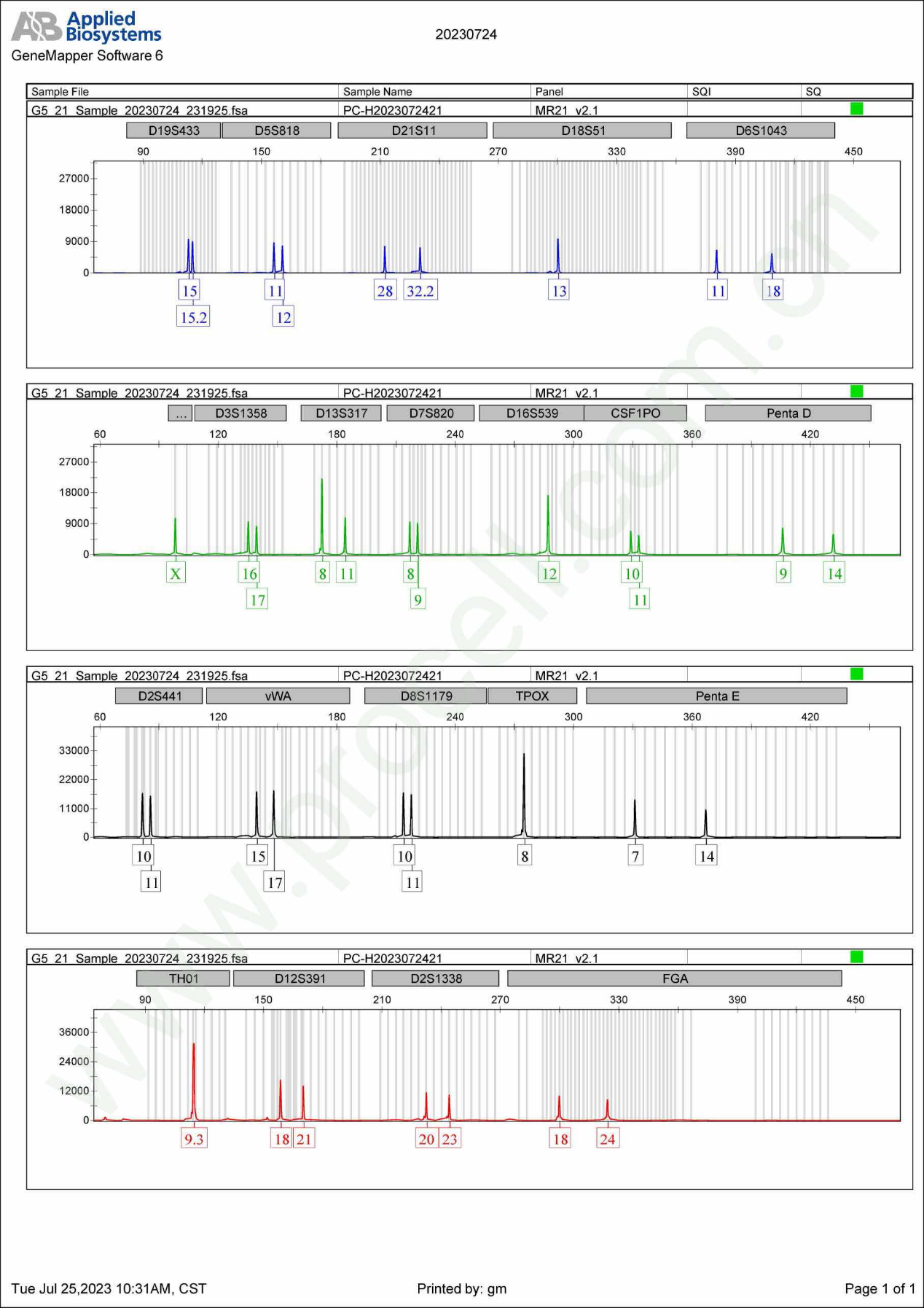

Supplement: Supplementary file 3 — Cell line STR [file 41420_2024_2098_MOESM3_ESM.docx]
